# Síndrome do Seio Carotídeo Associada a Carcinoma Ex-Adenoma Pleomórfico: Qual o Papel da Estimulação Cardíaca Artificial?

**DOI:** 10.36660/abc.20240026

**Published:** 2024-07-23

**Authors:** Vanessa Sanson Lani, Jorge Elias, Márcio Augusto Silva, Helena Alves de Andrade, Elisama Pimentel Zamian Cotias, Fernando Luiz Torres Gomes

**Affiliations:** 1 Hospital Universitário Cassiano Antonio Moraes Vitória ES Brasil Hospital Universitário Cassiano Antonio Moraes (HUCAM), Vitória, ES – Brasil; 2 Vitoria Apart Hospital Serra ES Brasil Vitoria Apart Hospital – Serviço de Eletrofisiologia, Serra, ES – Brasil; 3 Universidade Federal do Espirito Santo Departamento de Fisiologia Cardiovascular Vitoria ES Brasil Universidade Federal do Espirito Santo – Departamento de Fisiologia Cardiovascular, Vitoria, ES – Brasil

**Keywords:** Seio Carotídeo, Síncope, Neoplasias de Cabeça e Pescoço, Adenoma Pleomorfo, Estimulação Cardíaca Artificial

## Introdução

A síndrome do seio carotídeo (SSC) é definida como síncope ou pré-síncope associada a no mínimo 3 segundos de assistolia (resposta cardioinibitória) e/ou queda de pelo menos 50 mmHg na pressão arterial sistólica (resposta vasodepressora) em resposta à estimulação do seio carotídeo.^1^ Foi reconhecida pela primeira vez por Weiss e Baker em 1933, desde então, há relatos da associação entre massas cervicais e ocorrência de síncope.^2^ Dentre os causadores, encontra-se o carcinoma ex-adenoma pleomórfico (CXAP), neoplasia maligna rara que se desenvolve a partir de um adenoma pleomórfico primário ou recorrente. Representa 3,6% de todas as neoplasias das glândulas salivares e 11,6% de todas as neoplasias malignas destas glândulas. A SSC causada por massas cervicais é decorrente da compressão e invasão do seio carotídeo e ramos nervosos. Tal síndrome possui 3 subtipos: cardioinibitória, vasodepressora e mista.^3^ Um quarto tipo "resposta cerebral", foi previamente descrito na literatura, em que a compressão direta da artéria carótida causa isquemia cerebral ipsilateral levando a convulsões.^2^

## Apresentação do caso

**Figura 1:**
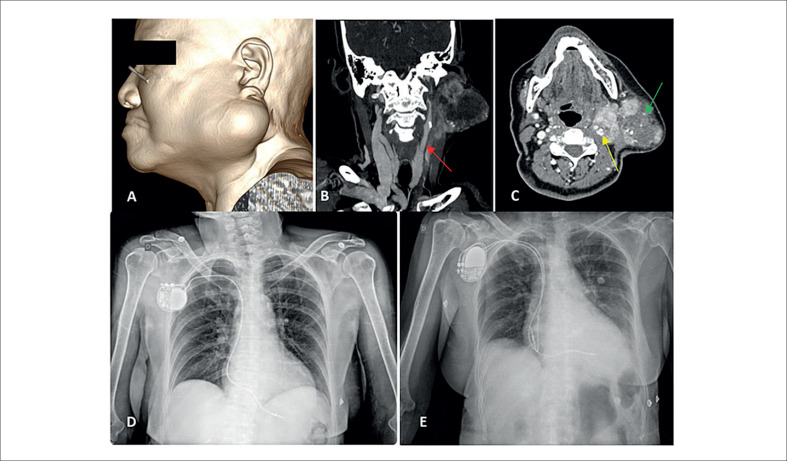
(A) Massa cervical volumosa localizada na região supra-hioidea, à esquerda. (B e C) Tomografia computadorizada de pescoço mostrando formação expansiva com densidade de partes moles e realce heterogêneo, áreas de necrose e focos de calcificação (seta verde), acometendo a parótida esquerda com abaulamento local e acometimento do espaço parafaríngeo, associada a múltiplas linfonodomegalias. A seta vermelha aponta para a carótida comum esquerda e a seta amarela para a bifurcação carotídea (interna e externa). (D) RX de tórax em AP após implante de MP unicameral. (E) RX de tórax com MP bicameral RDR.

Mulher, 69 anos, ex-tabagista há mais de 20 anos. Portadora de adenoma pleomórfico há 18 anos, foi conduzida inicialmente em tratamento conservador, porém, houve perda de seguimento clínico. Internada devido a síncopes recorrentes, sem pródomos, de início há 2 meses desencadeados por decúbito lateral esquerdo e rotação do pescoço à esquerda e com melhora após retorno da posição cervical no plano frontal. Apresentava massa cervical na região supra-hioide à esquerda de importante volume, indolor (Figura 1). Durante a internação apresentou episódios de bradicardia sinusal com frequência cardíaca (FC) de até 25 bpm, visualizadas em monitor cardíaco, com boa resposta a atropina. Como tratamento imediato foi implantado marca-passo (MP) provisório transvenoso com posterior inserção de MP definitivo unicameral (Medtronic) em modo VVI, 60 pulsos por minuto (Figura 1). A decisão de implantar um MP monocameral ventricular foi motivada pelo agravamento da condição clínica da paciente naquele momento, sem considerar a possibilidade de que o tipo de MP poderia interferir de forma desfavorável na evolução da síncope reflexa cardioinibitória. O ecocardiograma e a tomografia de crânio não apresentaram alterações. A tomografia cervical mostrou formação expansiva acometendo parótida esquerda, de 7,9 x 6,7 x 6,4 cm, com abaulamento local e acometimento do espaço parafaríngeo (Figura 1).

Inicialmente o implante de MP estabilizou a frequência cardíaca. Porém, a paciente evoluiu com novos sintomas de cefaleia, insônia, tontura e fraqueza. Houve também recorrência da síncope. Os episódios de mal-estar eram associados a sudorese profusa e náuseas e registro de ritmo de MP ventricular com condução ventrículo-atrial, quadro agora compatível com Síndrome do Marca-passo (SM). Dessa forma, por meio de contato com a equipe de eletrofisiologia, optou-se pela troca do dispositivo para sistema dupla-câmara, Gerador Medtronic modelo ATTESTA ATDR01 (DDDR), com função "*Rate-Drop Response*" (RDR) (Figura 1), o que resultou em remissão imediata dos sintomas.

**Figura 2:**
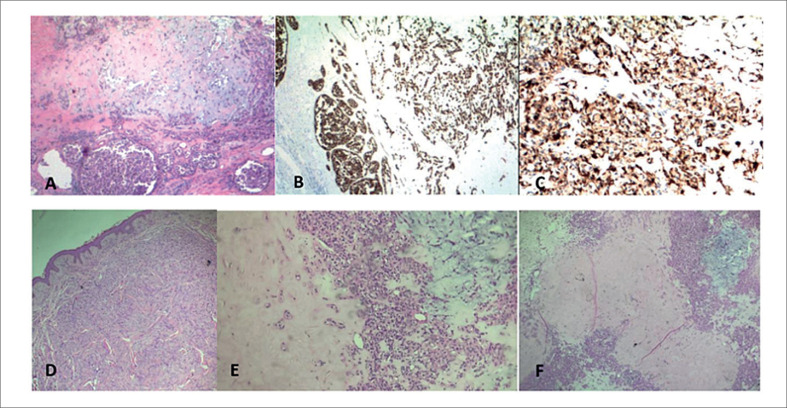
Estudo Imuno-Histoquímico mostrando neoplasia constituída por células epitelioides com citoplasma eosinofílico e núcleos regulares, dispostas em cordões ou blocos sólidos. (A). Áreas de esclerose estromal e áreas mixoides. Há células plasmocitoides de aspecto mioepitelial, com acúmulo de mucina. O estudo imuno-histoquímico revelou positividade para citoqueratina (B), proteína p63, calponina (focal) e S-100 (C). Tumor invadindo a derme (D), presença de células infiltrativas (E) e região mixoma do tumor (F). Esses achados são consistentes com neoplasia do tipo glândula salivar com áreas celulares de diferenciação mioepitelial, sendo as principais possibilidades o adenoma pleomórfico variante celular com predomínio mioepitelial e o carcinoma mioepitelial ex-adenoma pleomórfico.

A biópsia excisional da massa com estudo de imunohistoquímica revelou carcinoma ex-adenoma pleomórfico (Figura 2). Iniciou-se radioterapia, entretanto, não apresentava "*status performance"* para tratamento sistêmico com quimioterapia, necessitando de cuidados paliativos exclusivos. Retornou para consulta após 3 meses do implante do MP RDR, em ritmo sinusal próprio, negando episódios de síncope ou outros sintomas de hipofluxo cerebral. Estava em uso de hidralazina e clonazepam. Três dias após a avaliação internou-se por astenia, oligúria e hiporexia atribuídas a quadro séptico secundário a pneumonia evoluindo a óbito.

## Discussão

O adenoma pleomórfico consiste em uma massa indolor, de crescimento lento, dentro da parótida, áreas submandibulares ou cavidade bucal. A incidência de transformação maligna aumenta com a duração do tumor, sendo cerca de 10% para aqueles com mais de 15 anos de duração.^4^ A compressão e invasão do seio carotídeo e ramos nervosos (como nervo de Hering) pelo adenoma, leva a estimulação neural e ativação dos barorreceptores locais,^5^ desencadeando reflexo neurocardiogênico exagerado (cardioinibitória, vasodepressora ou mista) após a compressão do seio carotídeo.

O seio carotídeo é um componente importante de um sistema de controle neural responsável pela frequência cardíaca e pela homeostase da pressão arterial. A resposta hemodinâmica à distorção do seio carotídeo é a base da SSC. Essa resposta vagal é eliminada pela atropina,^6^ confirmando o diagnóstico da paciente em questão.

A estimulação cardíaca é geralmente bem-sucedida na prevenção de sintomas graves atribuíveis a SSC.^6^ Todavia, o implante de MP pode levar a complicações como a SM, que é a ocorrência de sintomas causados pela perda de sincronia AV, incluindo fadiga, dispneia, dor no peito, dor de cabeça e pulsações no pescoço. Pode haver dissociação AV ou condução VA 1:1 comprometendo a contração ventricular e desencadeando uma dissincronia AV. A condução retrógrada (VA), resulta em contração atrial enquanto as válvulas mitral e tricúspide estão fechadas.^7^ Essa entidade é mais frequente em pacientes com boa função cardíaca, sem distúrbio de condução atrioventricular avançado, e, preferencialmente, com estimulação do tipo VVI,^8,9^ como no caso relatado. Dessa maneira, foi realizada a troca de MP para dupla-câmara com função "*Rate-Drop Response*" (RDR), modo indicado para síncopes recorrentes por hipersensibilidade do seio carotídeo (forma cardioinibitória).^13^ A incorporação desse algoritmo programável que possibilita uma "resposta de queda da frequência cardíaca" é projetado para reconhecer alterações da frequência cardíaca habitualmente associadas a eventos vasovagais iminentes e desencadear um período autolimitado de estimulação bicameral com uma frequência mais elevada.^10^

Morley et al. observaram persistência dos sintomas em alguns pacientes utilizando MP ventricular (VVI) a despeito do normofuncionamento do dispositivo e se mostraram favoráveis à indicação de estimulação com MP dupla câmara em todos os pacientes portadores de SSC.^9^ Apesar de a associação entre SSC e disfunção do nó sinusal, síncope vasovagal ou hipotensão postural ser um limitador evidente no manejo terapêutico desses pacientes com tumor cervical, o MP dupla câmara, com algoritmo RDR, mostra-se efetivo na prevenção da recorrência dos eventos sincopais.^11^

Como tratamento alternativo, nos casos em que o MP não é possível, Takahiro et al. descreveu um caso cuja SSC induzida por câncer de pescoço foi efetivamente tratada com derivados de xantina, evitando a necessidade de MP enquanto o tumor era tratado. A explicação sugerida é um antagonismo competitivo dos derivados de xantinas na ativação periférica do quimiorreceptor de adenosina e do receptor endógeno de adenosina. Isso resulta em redução do efeito cronotrópico e dromotrópico negativos induzido pela adenosina. Os derivados da xantina também podem suprimir a resposta vasodepressora da adenosina.^12^

Uma questão que se mostra pertinente é se o MP, provisório ou definitivo, em alguns casos específicos, poderia desempenhar apenas papel adjuvante no tratamento até a extração do tumor e consequente resolução da compressão tumoral. Não existem dados na literatura que sinalizem que nessas situações seria possível considerar o explante do sistema com segurança.

Assim, a SSC é uma complicação rara e relevante dos tumores de cabeça e pescoço decorrente da hiperexcitação de barorreceptores. O tratamento de escolha consiste em implante de MP definitivo. Contudo, esse dispositivo pode levar a SM, condição que pode ser eficazmente tratada pela utilização de MP bicameral com função "*Rate-Drop Response*".
