# Supplementary material for: Síndrome do Seio Carotídeo Associada a Carcinoma Ex-Adenoma Pleomórfico: Qual o Papel da Estimulação Cardíaca Artificial?
Source: Arq Bras Cardiol. 2024 Jul 23;121(7):e20240026. [Article in Portuguese] doi: 10.36660/abc.20240026 (PMC11341206; doi:10.36660/abc.20240026)
Supplement: Translation

# Translation: Carotid Sinus Syndrome Associated with Carcinoma Ex-Pleomorphic Adenoma: What Is the Role of Artificial Cardiac Stimulation?

**DOI:** 10.36660/abc.20240026i

**Authors:** Vanessa Sanson Lani, Jorge Elias, Márcio Augusto Silva, Helena Alves de Andrade, Elisama Pimentel Zamian Cotias, Fernando Luiz Torres Gomes

**Affiliations:** 1 Vitória ES Brazil Hospital Universitário Cassiano Antonio Moraes (HUCAM), Vitória, ES – Brazil; 2 Serra ES Brazil Vitoria Apart Hospital – Serviço de Eletrofisiologia, Serra, ES – Brazil; 3 Vitoria ES Brazil Universidade Federal do Espirito Santo – Departamento de Fisiologia Cardiovascular, Vitoria, ES – Brazil

**Keywords:** Carotid Sinus, Syncope, Head and Neck Neoplasms, Adenoma Pleomorphic, Cardiac Pacing Artificial

## Introduction

Carotid sinus syndrome (CSS) is defined as syncope or pre-syncope associated with at least 3 seconds of asystole (cardioinhibitory response) and/or a drop of at least 50 mmHg in arterial systolic blood pressure (vasodepressor response) in response to stimulation of the carotid sinus.^1^ First recognized by Weiss and Baker in 1933, there have been since then reports of the association between neck masses and the occurrence of syncope.^2^ Among its causes is carcinoma ex-pleomorphic adenoma (CXAP), a rare malignant neoplasm that develops from a primary or recurrent pleomorphic adenoma. It represents 3.6% of all salivary gland neoplasms and 11.6% of all malignant neoplasms of these glands. CSS caused by neck masses is due to compression and invasion of the carotid sinus and nerve branches. This syndrome has 3 subtypes: cardioinhibitory, vasodepressor, and the mixed type.^3^ A fourth type of "cerebral response" has been previously described in the literature, in which direct compression of the carotid artery causes ipsilateral cerebral ischemia leading to seizures.^2^

## Case description

We present a 69-year-old woman, ex-smoker for over 20 years, diagnosed with pleomorphic Adenoma for 18 years. The patient was receiving conservative approach treatment, but short of a consistent clinical follow-up. She was hospitalized due to recurrent syncope, without prodromes, that began 2 months before hospitalization, triggered by left lateral decubitus and rotation of the neck to the left and with improvement after returning to the cervical position in the frontal plane.

She presented a significant voluminous, painless cervical mass, in the suprahyoid region, on the left (Figure 1). During hospitalization, she presented episodes of sinus bradycardia with a heart rate (HR) of up to 25 bpm, visualized on a cardiac monitor, and a good response to Atropine. As an immediate treatment, a temporary transvenous pacemaker (PM) was implanted with subsequent implantation of a definitive single-chamber PM (Medtronic) in VVI mode at 60 pulses per minute (Picture 1). The decision to implant a single-chamber ventricular PM was driven by the worsening of the patient's clinical condition without considering the possibility that this type of PM could unfavorably interfere with the evolution of the cardioinhibitory reflex syncope. Echocardiogram and head tomography showed no changes. The cervical tomography showed an expansive formation affecting the left parotid, measuring 7.9 x 6.7 x 6.4 cm, with local bulging and involvement of the parapharyngeal space (Figure 1).

**Figure 1:** (A) Large neck mass located in the suprahyoid region, on the left. (B and C) CT-scan of the neck showing expansive formation with soft tissue density and heterogeneous enhancement, areas of necrosis, and foci of calcification (green arrow), affecting the left parotid with local bulging and involvement of the parapharyngeal space, associated with multiple lymph node enlargement. Red arrow points to the left common carotid artery and yellow arrow to the carotid bifurcation (internal and external). (D) AP chest X-ray after single-chamber PM implantation. (E) Chest X-ray with bicameral PM RDR.

Initially, the pacemaker implant stabilized the heart rate. However, the patient developed new symptoms such as headache, insomnia, dizziness, and weakness. There was also a recurrence of syncope. The episodes of malaise were associated with profuse sweating and nausea and recording of ventricular PM rhythm with ventriculo-atrial conduction, a condition now compatible with pacemaker syndrome (MS). Therefore, through contact with the electrophysiology team, it was hence opted the replacement of the device for a Medtronic model ATTESTA ATDR01 (DDDR), double-chamber system generator with "Rate-Drop Response" (RDR) function) (Figure 1), what resulted in an immediate reduction of symptoms.

Excisional biopsy of the mass with an immunohistochemistry study revealed carcinoma ex-pleomorphic adenoma (Figure 2). Radiotherapy was started, however, it did not present "performance status" for systemic treatment with chemotherapy, requiring exclusive palliative care. The patient returned for consultation 3 months after implantation of the PM RDR, presenting proper sinus rhythm, lacking episodes of syncope, or other symptoms of cerebral hypoflow. She was on hydralazine and clonazepam.

**Figure 2:** Immunohistochemical study showing a neoplasm consisting of epithelioid cells with eosinophilic cytoplasm and regular nuclei, arranged in cords or solid blocks. (A). Areas of stromal sclerosis and myxoid areas. There are plasmacytoid cells with a myoepithelial appearance, with an accumulation of mucin. The immunohistochemical study revealed positivity for cytokeratin (B), p63 protein, calponin (focal) and S-100 (C). Tumor invading the dermis (D), presence of infiltrative cells (E), and Myxoma region of the tumor (F). These findings are consistent with salivary gland-type neoplasia with cellular areas of myoepithelial differentiation, the main possibilities being cellular variant pleomorphic adenoma with myoepithelial predominance and myoepithelial carcinoma ex-pleomorphic adenoma.

Three days after the evaluation, she was hospitalized due to asthenia, oliguria, and hyporexia attributed to septic conditions secondary to pneumonia, which resulted in her death.

## Discussion

Pleomorphic adenoma consists of a painless, slow-growing mass within the parotid, submandibular areas or oral cavity. The incidence of malignant transformation increases with the duration of the tumor, being around 10% for those lasting more than 15 years.^4^ Compression and invasion of the carotid sinus and nerve branches (such as Hering's nerve) by the adenoma lead to neural stimulation and activation of local baroreceptors,^5^ triggering an exaggerated neurocardiogenic reflex (cardioinhibitory, vasodepressor, or mixed) after compression of the carotid sinus.

The carotid sinus is an important component of a neural control system responsible for heart rate and blood pressure homeostasis. The hemodynamic response to carotid sinus distortion is the basis of SSC. This vagal response is eliminated by atropine,^6^ confirming the diagnosis of the referred patient.

Cardiac pacing is generally successful in preventing severe symptoms attributable to SSC.^6^ However, PM implantation can lead to complications such as MS, which is the occurrence of symptoms caused by loss of AV synchrony, including fatigue, dyspnea, chest pain, headache, and throbbing in the neck. There may be AV dissociation or 1:1 VA conduction, compromising ventricular contraction and triggering AV dyssynchrony. Retrograde conduction (VA) results in atrial contraction while the mitral and tricuspid valves are closed.^7^ This distinct condition is more common in patients with good cardiac function, without advanced atrioventricular conduction disturbance, and, preferably, with VVI^8,9^ type stimulation, as in the case reported. For this very reason, the PM was switched to dual-chamber with the "Rate-Drop Response" (RDR) function, a mode indicated for recurrent syncope due to carotid sinus hypersensitivity (cardioinhibitory form)^13^. The embedding of this programmable algorithm that enables a "fall heart rate response" is designed to recognize heart rate changes typically associated with impending vasovagal events and trigger a self-limited period of bicameral stimulation at a higher rate.^10^

Morley et al. observed the persistence of symptoms in some patients using ventricular PM (VVI) despite the normal functioning of the device and were in favor of indicating stimulation with double chamber PM in all patients with SSC.^9^ Although the association between SSC and sinus node dysfunction, vasovagal syncope or postural hypotension is an obvious limitation in the therapeutic management of these patients with cervical tumors, double-chamber PM, with the RDR algorithm, is effective in preventing the recurrence of syncopal events.^11^

As an alternative treatment, in cases where PM is not possible, Takahiro et al. described a case whose neck cancer-induced SSC was effectively treated with xanthine derivatives, avoiding the need for PM while the tumor was treated.

The suggested explanation is a competitive antagonism of xanthine derivatives in the peripheral activation of the adenosine chemoreceptor and the endogenous adenosine receptor. This results in a reduction in the negative chronotropic and dromotropic effects induced by adenosine.^12^

An underlying issue that appears to be pertinent is whether PM, whether provisional or definitive, in some specific cases, could only play a side role in treatment until tumor extraction and consequent resolution of tumor compression. There are no data in the literature that indicate that in these situations it would be possible to safely consider explanting the system.

Thus, SSC is a rare and relevant complication of head and neck tumors resulting from baroreceptor hyperexcitation. The treatment of choice consists of permanent PM implantation. However, this device can lead to SM, a condition that can be effectively treated by using a bicameral PM with a "Rate-Drop Response" function.
